# Mechanism of lncRNA SNHG16 in oxidative stress and inflammation in oxygen-glucose deprivation and reoxygenation-induced SK-N-SH cells

**DOI:** 10.1080/21655979.2022.2026861

**Published:** 2022-02-16

**Authors:** Xiangyuan Cao, Jingjing Ma, Shaohua Li

**Affiliations:** a Department of Neurosurgery, Shanghai Tenth People’s Hospital, Clinical Medical College of Nanjing Medical University, Shanghai, China; b School of Medicine, Shanghai Tenth People’s Hospital, Tongji University, Shanghai, China; c Department of Orthopedics, Shanghai Tenth People’s Hospital, Clinical Medical College of Nanjing Medical University, Shanghai, China

**Keywords:** SNHG16, miR-421, xiap, oxygen-glucose deprivation and reoxygenation, oxidative stress injury, inflammation, ceRNA

## Abstract

Cerebral ischemia-reperfusion injury imposes a clinical challenge for physicians in the wake of ischemic stroke. Meanwhile, recent evidence has come to light eliciting the neuroprotective function of SNHG16 in cerebrovascular diseases. Accordingly, the current study sought to analyze the regulatory mechanism of long non-coding RNA small nucleolar RNA host gene16 (SNHG16) in oxidative stress (OS) injury and cell inflammation. Firstly, models of oxygen-glucose deprivation and reoxygenation (OGD/R) were established in SK-N-SH cells. Cell proliferation and apoptosis were appraised using cell counting kit-8 and flow cytometry. Additionally, SNHG16, X-linked inhibitor of apoptosis protein (XIAP), microRNA (miR-421), reactive oxygen species (ROS), lactate dehydrogenase (LDH), malondialdehyde (MDA), superoxide dismutase (SOD), tumor necrosis factor -α, interleukin (IL)-1β, and IL-10 expression patterns were determined. In addition, we determined and validated the subcellular localization of SNHG16 and the binding relationships between SNHG16 and miR-421, and miR-421 and XIAP. It was found that SNHG16 was poorly-expressed in OGD/R-treated cells. On the other hand, SNHG16 over-expression enhanced cell proliferation, inhibited apoptosis, and alleviated OS and cell inflammation. Furthermore, SNHG16 bound to miR-421 to facilitate the expression of XIAP. Up-regulation of miR-421 or down-regulation of XIAP could reverse the suppressive effects of SNHG16 on OS and cell inflammation. Collectively, our findings indicated that SNHG16 bound to miR-421 to facilitate XIAP expression, thus alleviating OS injury and inflammation in OGD/R-induced SK-N-SH cells.

## Introduction

Stroke remains one of the leading cerebrovascular diseases with high disability and mortality rates, negatively affecting the healthy lives of more than 13 million people worldwide [1]. Moreover, ischemic stroke represents the major subtype of stroke, accounting for appropriately 80% of all cases, while resulting from obstruction of the blood clot and subsequent oxygen and glucose deficits [2,3]. Meanwhile, reperfusion signifies the process of increasing blood supply to ischemic lesions but associated with a series of secondary injuries, such as oxidative stress (OS), local inflammation, and cell death, which is described as ischemia-reperfusion (I/R) injury [4]. The last few decades have greatly augmented our understanding of the molecular mechanism of cerebral I/R injury and treatments, ranging from hypothermia therapy to therapies targeting inflammation, activated platelet receptors, and calcium influx, all of which exhibit neuroprotective efficacy to a certain extent [3]. Nevertheless, there are limitations to current clinical management regimens, and advanced therapeutics remains to be investigated.

Long non-coding RNAs (lncRNAs), a class of ncRNAs with a length of more than 200 nt, are known to confer a regulatory role in brain development and I/R injury [5,6]. In a similar light, lncRNA small nuclear RNA host gene 16 (SNHG16), derived from chromosome 17q25.1, is well-documented for its oncogenic property in numerous types of malignancies [7,8]. Furthermore, recent studies have uncovered the ability of SNHG16 to protect neurons from ketamine neurotoxicity [9], while also undergoing targeting by dexmedetomidine to exert neuroprotective function [10]. What’s more, a prior study documented that SNHG16 was decreased in response to cerebral I/R injury, such that its supplementation could potently suppress I/R injury-induced cell apoptosis [11]. However, the underlying regulatory role of SNHG16 in OS and inflammation post-cerebral I/R injury requires further exploration.

The well-established competing endogenous RNA (ceRNA) mechanism refers to a regulatory network, wherein lncRNAs regulate the expressions of its downstream message RNA (mRNA) by sponging its target microRNAs (miRNA) [12]. Interestingly, SNHG16 was previously reported to function as a ceRNA in cerebral I/R injury *via* repression of miR-183 [13]. Furthermore, various miRNAs, representing short ncRNAs with a length of about 70 nt, are further involved in the epigenetic regulation of cerebral I/R injury, such as miR-211 and miR-30 c-5p [14,15]. Another such miRNA, namely miR-421, was recently indicated to be capable of potentiating neurotoxicity and neuronal death in Parkinson’s Disease [16]. In addition, miR-421 is aberrantly expressed in cerebral I/R injury, and further associated with cell apoptosis and oxidative stress post-cerebral I/R injury [17]. Herein, initial database prediction and dual-luciferase reporter assay in the current study indicated that X-linked inhibitor-of-apoptosis protein (XIAP) served as a target of miR-421. XIAP is further regarded as an endogenous inhibitor of caspases, which restricts the activation of pro-apoptosis proteins and the production of inflammasomes [18]. Additionally, XIAP has been indicated to play a suppressive role in infarct expansion, neurological dysfunction, and neuronal apoptosis, thereby participating in preconditioning treatment of cerebral I/R injury [19,20]. Nonetheless, the crosstalk of SNHG16 and miR-421/XIAP in cerebral I/R injury has not been reported at home or abroad.

Based on the preceding evidence, we hypothesized that SNHG16 acts as a ceRNA to regulate OS injury and inflammation post-cerebral I/R injury *via* modulation of the miR-421/XIAP axis. Accordingly, we established a cell model of oxygen-glucose deprivation/reperfusion (OGD/R) to mimic the cerebral I/R injury. In this way, the current study sought to uncover the therapeutic value of SNHG16 in OGD/R-induced OS injury and inflammation and provide novel targets for the efficacious treatment of post-cerebral I/R injury.

## Methods and Materials

### Ethics statement

The current study was carried out with the approval of the Ethics committee of Shanghai Tenth People’s Hospital.

### Cell culture and transfection

Referring to previously published literature [21], SK-N-SH cells (ATCC, Manassas, VA, USA) were placed in Dulbecco’s modified Eagle medium (DMEM) (Invitrogen, Carlsbad, CA, USA) (containing 10% fetal bovine serum and 100 U/mL penicillin/streptomycin) with humidified air of 37°C and 5% CO_2_ for 48 h. Next, the SK-N-SH cells were seeded into 6-well plates at a density of 6 × 10^4^ cells/cm^2^, followed by transfection with 2 μg pCDNA3.1-SNHG16 (Genecopoeia, Guangzhou, China), 100 nM miR-421 mimic (Thermo Fisher Scientific, Waltham, MA, USA), 50 nM si-XIAP-1 and si-XIAP-2 (Shanghai BGI Technology Co., LTD, Shanghai, China) and equal volumes of corresponding negative controls using the Lipofectamine 2000 reagent (Invitrogen, Carlsbad, CA, USA). Prior to OGD/R treatment, the cells were cultured for 48 consecutive h, and the transfection efficiency was analyzed by means of reverse transcription quantitative polymerase chain reaction (qRT-PCR). Subsequently, the transfected cells were deprived of the normal medium, rinsed with phosphate buffer saline (PBS) 3 times, and cultured in serum/glucose-free DMEM. Afterward, the cells were placed in a hypoxic chamber (containing 95% N_2_ and 5% CO_2_) and then cultured under normal conditions for 12 h to induce OGD/R injury. Simultaneously, control cells were cultured at 37°C with 5% CO_2_ in air without any special treatment.

### Cell viability assay

In accordance with previously published literature [22], cell viability of the above-mentioned cells was assessed using cell counting kit-8 (CCK-8) (Dojindo Laboratories, Kumamoto, Japan). Briefly, the cell suspension was seeded into 96-well plates (at a density of 5000 cells/well) and placed in a humidified incubator at 37°C with 5% CO_2_ in air. At the 0 h, 24 h, 36 h, 48 h, and 60 h time intervals, the plates were added with 10 μL CCK-8 solution, followed by 1 h-incubation at 37°C. Afterward, the absorbance values at a wavelength of 450 nm were measured using a microplate reader (Dojindo Laboratories).

### Flow cytometry

Referring to previously published literature [21], cell apoptosis of the above-mentioned cells was assessed using flow cytometry with the help of Annexin V/PI kits (Biouniquer, Beijing, China). Briefly, the cells were harvested and rinsed with precooled PBS twice. Next, the cells were resuspended in the binding buffer and stained with Annexin V-FITC and propidium iodide for 30 min in conditions void of light at room temperature. Flow cytometry was performed using the Coulter® EPICS XL instrument (Beckman Coulter, Fullerton, CA, USA). Apoptosis rate was analyzed with the Flowing version 2.5.1 software (Turku Bioscience, Turku, Finland) and Origin version 8 software (OriginLab, Northampton, MA, USA).

### Reactive oxygen species (ROS) production assay

Transfected cells were added with 10 μM DCFH-DA (Beyotime Institute of Biotechnology, Beijing, China), followed by immersion in a water bath at 37°C for 30 min. In accordance with previously published literature [23], ROS activity was analyzed using the ImageJ version 6.0 software (National Institutes of Health, Bethesda, MD, USA). A total of 5 randomly selected wells were observed under a fluorescence microscope (Nikon, Tokyo, Japan).

### Key protein activity assay

Referring to previously published literature [24], cell supernatants of different treatment groups were collected. In accordance with the manufacturer’s protocol, lactate dehydrogenase (LDH) activity in cell supernatant was detected using LDH assay kits (ab102526, Abcam, Cambridge, UK), while malondialdehyde (MDA) levels were measured using lipid peroxidation (MDA) assay kits (ab118970, Abcam) and superoxide dismutase (SOD) activity was measured using a SOD assay (500–100-K, R&D System, Inc., Minneapolis, MN, USA).

### Enzyme-linked immunosorbent assay

In accordance with previously published literature [25], levels of tumor necrosis factor (TNA)-α and interleukin (IL)-1β were determined using TNF alpha Human ELISA kits (KHC3013, Thermo Fisher Scientific) and Human IL-1 beta Quantikine ELISA kits (201-LB, R&D SYSTEMS), respectively. Additionally, IL-10 levels were determined with IL-10 Human ELISA kits (Thermo Fisher Scientific) following the manufacturer’s instructions.

### qRT-PCR

Referring to previously published literature [26], total RNA content was extracted from the above-mentioned cells using the TRlzol reagent (Invitrogen). The obtained RNA and miRNA were reverse-transcribed into the complementary DNA using iScript Cdna synthesis kits (Bio-Rad, Hercules, CA, USA) and TaqMan™ MicroRNA reverse transcription kits (94,366,596, Thermo Fisher Scientific), respectively. Real-time quantitative PCR was subsequently performed using SYBR™ Green PCR (4,309,155, Thermo Fisher Scientific) and an ABI 7500 fast real-time PCR system (ABI, Inc., Foster City, CA, USA). U6 and GAPDH were adopted as the endogenous controls, and the relative gene expression was measured using the 2^−ΔΔCt^ method. Primers of qRT-PCR were shown in Table 1.

**Table 1.** qPCR primers

Gene	Forward Primer (5ʹ-3ʹ)	Reverse Primer (5ʹ-3ʹ)
LncRNA SNHG16	GCGTTCTTTTCGAGGTCGGCCG	TGACGGTAGTTTCCCAAG
miR-421	GCCGAGCGCGGGUUAAUUACA	CTCAACTGGTGTCGTGGA
XIAP	CAAGAGAAGATGACTTTTAAC	TTAAGACATAAAAATTT
U6	CTCGCTTCGGCAGCACAT	AACGCTTCACGAATTTGCGT
GAPDH	GCACCGTCAAGGCTGAGAAC	TGGTGAAGACGCCAGTGGA

### Bioinformatics analysis

Subcellular localization of SNHG16 was predicted using the LncATLAS website (http://lncatlas.crg.eu) [27]. Additionally, the downstream target miRNAs of lncRNA SNHG16 were analyzed with the help of the LncBase v.2 website (http://carolina.imis.athena-innovation.gr/diana_tools/web/index.php?r=lncbasev2%2Findex) [28] and the StarBase website (http://starbase.sysu.edu.cn/) [29]. Furthermore, the target genes of miR-421 were analyzed using the TargetScan (http://www.targetscan.org/) [30], RNA22 (https://cm.jefferson.edu/rna22/Interactive/) [31], StarBase and miRTarBase (http://mirtarbase.cuhk.edu.cn/php/index.php) [32].

### Nuclear/Cytosol fractionation assay

PARIS kits (AM1921, Thermo Fisher Scientific, Waltham, MA, USA) were adopted for the separation of nuclear and cytoplasmic components. Referring to previously published literature [33], SK-N-SH cells were collected, rinsed with PBS, and placed on ice. Subsequently, the cells were resuspended with an ice-cold cell fractionation buffer and cultured on ice for 10 min. Following centrifugation, the supernatant was discarded and the remaining part was nuclear particles. The TRIzol reagent (Invitrogen) was adopted for the extraction of nuclear and cytoplasmic RNA, and the M-MLV assay kits (28,025,013, Thermo Fisher Scientific) were utilized for reverse transcription. SNHG16 expression patterns in the nuclei and cytoplasm were detected using qRT-PCR, with serving GAPDH and U6 as controls.

### Dual-luciferase reporter assay

Referring to previously published literature [34], the wild type (WT) SNHG16 sequence or WT XIAP mRNA 3ʹUTR fragments containing the miR-421 binding sites were amplified and inserted into pmirGLO dual-luciferase miRNA target expression vectors (Promega, Madison, WI, USA) to construct pmirGLO-SNHG16-WT and pmirGLO-XIAP-WT vectors. Mutation (MUT) of SNHG16 or XIAP 3ʹUTR containing the miR-421 binding sites was processed using a GeneArtTM site-directed mutagenesis system (A14604, Thermo Fisher Scientific). Subsequently, SNHG16-MUT or XIAP 3ʹUTR-MUT were inserted into the pmirGLO to construct pmirGLO-SNHG16-MUT and pmirGLO-XIAP-MUT. The aforementioned vectors and miR-421 mimic or mimic NC were co-transfected into SK-N-SH cells, followed by 48-h of incubation. Afterward, luciferase activity was measured using a dual-luciferase reporter determination system (Promega).

### RNA pull-down assay

In accordance with previously published research [35], SK-N-SH cells (N = 6 × 10^6^) were cultured at 37°C for 24 h, followed by transfection with biotinylated miR-421 and its control (Guangzhou RiboBio Co., Ltd, Guangzhou, China). Following OGD/R treatment and 48-h of oxygen enrichment, total RNA content was extracted using the TRlzol reagent (Invitrogen). Subsequently, the biotinylated RNA was absorbed with M-280 chain-typed magnetic beads (Invitrogen), and then the magnetic beads were rinsed with hypersaline buffer (containing 1% Triton X-100, 0.1% SDS, 20 mM Tris-HCl (pH 8.0), 2 mM ethylene diamine tetraacetic acid; 500 mM NaCl). Lastly, RNA expression patterns were detected using qRT-PCR.

### Western blot assay

Referring to previously published literature [25], total protein content was extracted from the above-mentioned cells using Radio Immunoprecipitation Assay lysis containing protease and phosphatase inhibitors, and the concentration was quantified with the help of bicinchoninic acid protein detection kits (Nanjing Jiancheng Bioengineering Institute, Nanjing, Jiangsu, China). Subsequently, 50 μg protein was isolated in sodium dodecyl sulfate gel and transferred to polyvinylidene fluoride membranes. Next, the membranes were blockaded with Tris buffer containing 0.05% Tris Buffered Saline Tween and 5% skim milk and incubated with the primary antibodies XIAP (dilution ratio of 1:1000; ab21278; Abcam) and β-actin (dilution ratio of 1:5000; ab6276; Abcam) at 4°C overnight, followed by incubation with the secondary antibody IgG (dilution ratio of 1:5000; ab205719; Abcam) at room temperature for 1 h. The antigen/antibody complex was detected with an enhanced chemiluminescence reagent. The relative expression patterns of proteins were analyzed using the Image J software (National Institutes of Health, Bethesda, ML, USA).

### Statistical analysis

Data analyses and graphing were processed using the SPSS21.0 statistical software (IBM Corp, Armonk, NY, USA) and GraphPad Prism 8.0 software (GraphPad Software Inc., San Diego, CA, USA). Measurement data were exhibited as mean ± standard deviation (SD) and conformed to normal distribution and homogeneity of variance. The *t* test was adopted for pairwise comparisons. One-way or two-way analysis of variance (ANOVA) was appointed for multi-group comparisons, followed by Tukey’s multiple comparison test for posttest data. *P* value was obtained from two-tailed tests, and a value of *P* < 0.05 was regarded statistically significant.

## Results

The current study was initiated with the establishment of cerebral I/R cell models in SK-N-SH cells using OGD/R treatment and transfection with SNHG16 over-expression vector into SK-N-SH cells. Subsequent findings revealed that SNHG16 was poorly-expressed in OGD/R-treated SK-N-SH cells, whereas SNHG16 over-expression alleviated OGD/R-induced OS injury and cell inflammation. Additionally, we explored the downstream mechanism of SNHG16 and found that SNHG16 as a ceRNA bound to miR-421 to up-regulate the expression of XIAP, thus alleviating OGD/R-induced OS injury and cell inflammation.

### SNHG16 was poorly-expressed upon OGD/R and its overexpression attenuated OS injury and cell inflammation post-OGD/R

**Figure 1.**
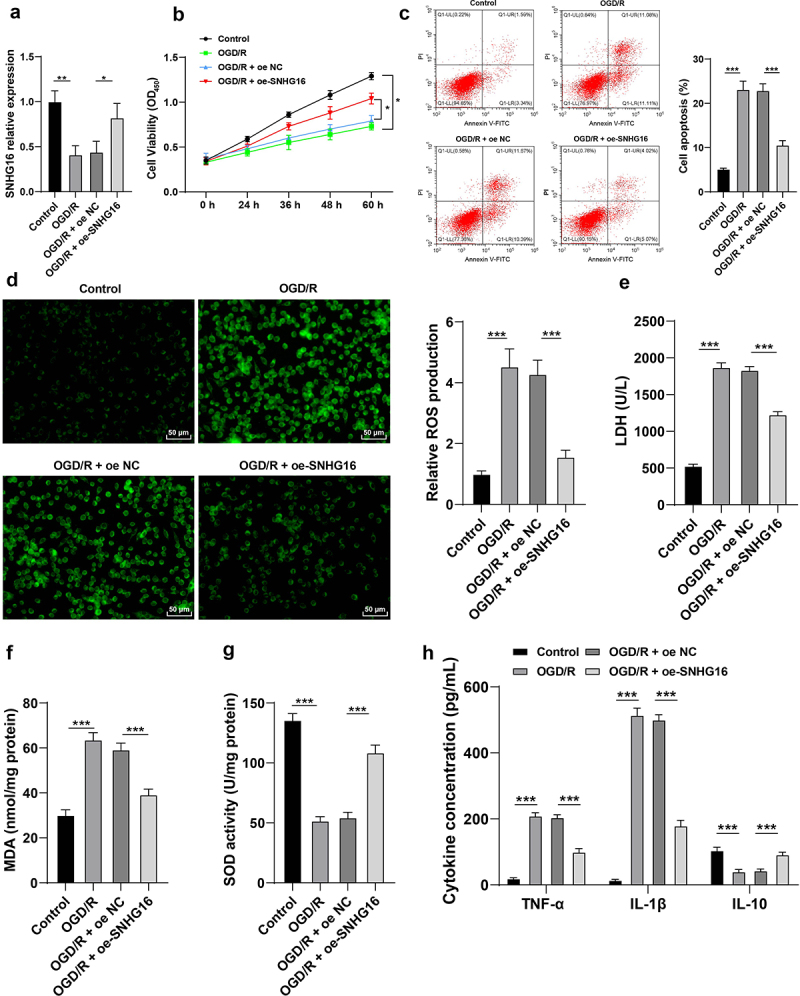
SNHG16 was poorly expressed upon OGD/R and its overexpression attenuated OSI and cell inflammation post-OGD/R. The cerebral I/R cell model was established with normally cultured SK-N-SH cells as the control. a: SNHG16 expression detected via qRT-PCR; b: Cell proliferation assessed via CCK-8; c: Cell apoptosis assessed via flow cytometry; d: ROS level measured via fluorescence labeling; e-g: LDH, MAD, and SOD activities determined via corresponding assay kits; h: TNF-α, IL-1β, and IL-10 concentrations determined via ELISA. Cell experiments were performed 3 times independently and data were exhibited as mean ± SD. Data in figures A, C, D, E, F, and G were analyzed using one-way ANOVA and data in figures B and H were analyzed using two-way ANOVA, followed by Tukey’s multiple comparison test. * *P* < 0.05; ** *P* < 0.01; ***P < 0.001.

Existing evidence indicates that SNHG16 is weakly-expressed in cerebral I/R injury, whereas its overexpression could promote cell survival and inhibit apoptosis [11]. Accordingly, cerebral I/R cell models were established via OGD/R treatment in human neuroblastoma cells SK-N-SH. It was found that SNHG16 expression levels were lower in OGD/R-treated cells compared to those in control cells (*P* < 0.05, Figure 1a). Presumably, SNHG16 plays a crucial role in the OGD/R injury of SK-N-SH cells. Following OGD/R treatment, SK-N-SH cell proliferation potential was abated (*P* < 0.05, Figure 1b), apoptosis rate was elevated (*P* < 0.05, Figure 1c), ROS, LDH and MDA levels were increased (*P* < 0.05, Figure 1d-f), SOD activity was diminished (*P* < 0.05, Figure 1g), TNF-α and IL-1β levels were augmented, while IL-10 concentration was found to be decreased (*P* < 0.05, Figure 1h). To further confirm whether SNHG16 could regulate OS injury and cell inflammation post-OGD/R, OGD/R-treated SK-N-SH cells were treated with pcDNA3.1-NC and pcDNA3.1-SNHG16 (*P* < 0.05, Figure 1a). Altogether, the aforementioned findings indicated that SNHG16 overexpression attenuated OGD/R-induced OS injury and cell inflammation (*P* < 0.05, Figure 1b-h).

### SNHG16 inhibited miR-421 expression

**Figure 2.**
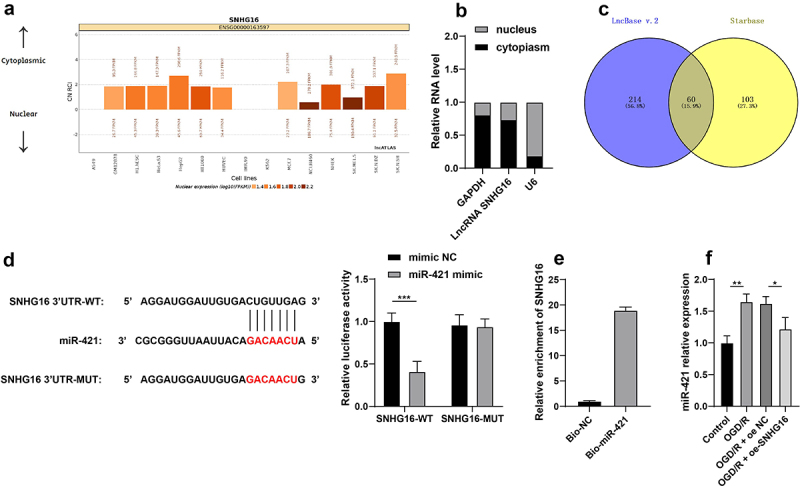
SNHG16 inhibited miR-421 expression. a: Subcellular localization of SNHG16 predicted via the LncATLAS website (http://lncatlas.crg.eu/); b: Subcellular localization of SNHG16 testified via the nuclear/Cytosol fractionation assay; c: Downstream miRNAs of SNHG16 predicted via the LncBase v.2 and Starbase websites, and the Venn diagram was plotted; d-e: The binding of SNHG16 and miR-421 verified via dual-luciferase reporter assay (d) and RNA pull-down assay (e); F: miR-421expression detected via qRT-PCR. Cell experiments were performed 3 times independently and data were exhibited as mean ± SD. Data in figure E were analyzed using *t* test. Data in figure F were analyzed using one-way ANOVA and data in figure D were analyzed using two-way ANOVA, followed by Tukey’s multiple comparison test. * *P* < 0.05; ** *P* < 0.01; ***P < 0.001.

To further probe the molecular mechanism of SNHG16 in cerebral I/R injury, the LncATLAS website (http://lncatlas.crg.eu/) was utilized to predict the subcellular localization of SNHG16, which indicated that SNHG16 was located in the cytoplasm (Figure 2a). Subsequent nuclear/cytosol fractionation assay further confirmed that SNHG16 was primarily expressed in the cytoplasm (Figure 2b), suggesting that SNHG16 could potentially act as a ceRNA to play a role in cerebral I/R injury. Accordingly, downstream miRNAs of SNHG16 were predicted using the LncBase v.2 and StarBase websites and their intersections were identified (Figure 2c). Then, we focused on miR-421. Prior research has evidenced that miR-421 is highly expressed in cerebral I/R injury, such that down-regulation of miR-421 can alleviate cerebral I/R injury [17]. Furthermore, the binding relationship of SNHG16 and miR-421 was validated by means of dual-luciferase reporter assay and RNA pull-down assay (*P* < 0.05, Figure 2d, e). It was observed that miR-421 was up-regulated in OGD/R-treated cells, whereas SNHG16 over-expression diminished miR-421 expression levels in OGD/R-treated cells (*P* < 0.05, figure 2f). Together, these findings indicated that SNHG16 could inhibit the expression of miR-421.

### miR-421 up-regulation partly reversed the alleviating role of SNHG16 in OS injury and cell inflammation post-OGD/R

**Figure 3.**
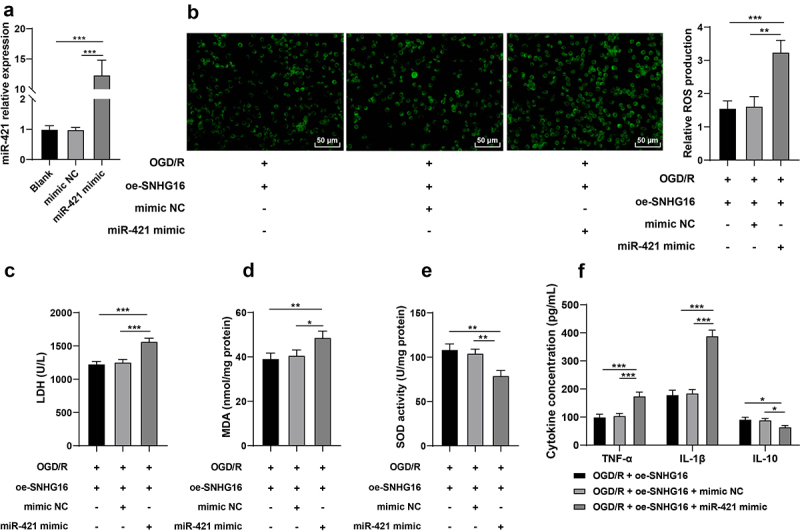
miR-421 upregulation partly reversed the alleviating role of SNHG16 in OSI and cell inflammation post-OGD/R. miR-421 mimic was transfected into oe-SNHG16-treated SK-N-SH cells with mimic NC as the control. a: The transfection efficiency of miR-421 detected via qRT-PCR; b: ROS level measured via fluorescence labeling; c-e: LDH, MAD and SOD activities determined via corresponding assay kits; f: TNF-α, IL-1β, and IL-10 concentrations determined via ELISA. Cell experiments were performed 3 times independently and data were exhibited as mean ± SD. Data in figures A, B, C, D, and E were analyzed using one-way ANOVA and data in figure F were analyzed using two-way ANOVA, followed by Tukey’s multiple comparison test. * *P* < 0.05; ** *P* < 0.01; ***P < 0.001.

Furthermore, oe-SNHG16-treated SK-N-SH cells were transfected with miR-421 mimic (*P* < 0.05, Figure 3a). Subsequent results showed that compared with the oe-SNHG16 group, miR-421 up-regulation facilitated the production of ROS levels (*P* < 0.05, Figure 3b), enhanced the activity of LDH and MDA (*P* < 0.05, Figure 3c, d), reduced the SOD activity (*P* < 0.05, Figure 3e), elevated concentrations of TNF-α and IL-1β and diminished that of IL-10 (*P* < 0.05, figure 3f). Collectively, these findings indicated that miR-421 up-regulation reversed the alleviating role of SNHG16 in OS injury and cell inflammation post-OGD/R to some extent.

### miR-421 inhibited XIAP expression

**Figure 4.**
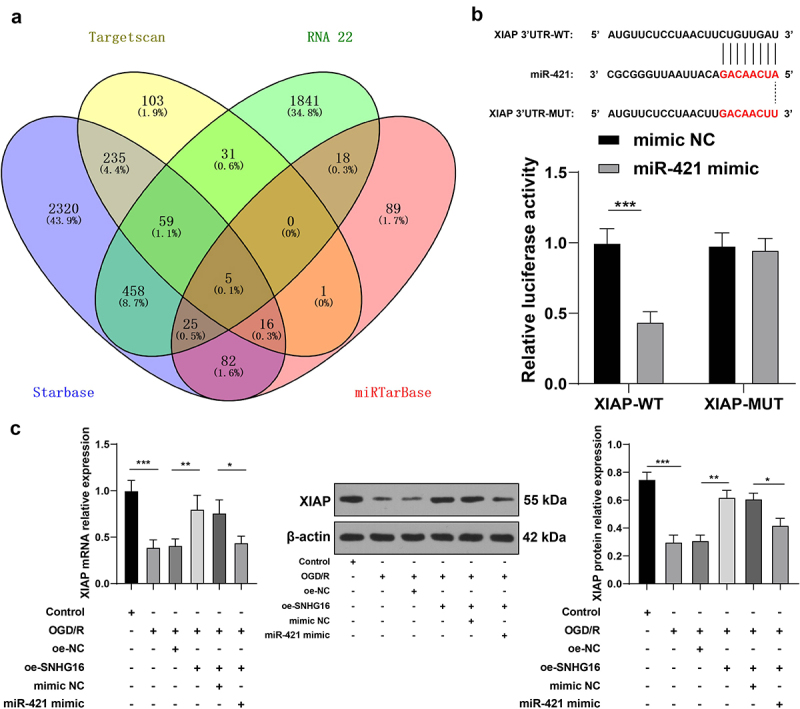
miR-421 inhibited XIAP expression. a: Downstream target genes of miR-421 predicted via the Starbase, TargetScan, RNA22 and miRTarBase websites; b: The binding of miR-421 and XIAP testified via the dual-luciferase reporter assay; c: mRNA and protein levels of XIAP detected via qRT-PCR and Western blotting. Cell experiments were performed 3 times independently and data were exhibited as mean ± SD. Data in figure C were analyzed using one-way ANOVA and data in figure B were analyzed using two-way ANOVA, followed by Tukey’s multiple comparison test. * *P* < 0.05; ** *P* < 0.01; ***P < 0.001.

To further analyze the downstream mechanism of miR-421, we predicated the downstream target genes of miR-421through the StarBase, TargetScan, RNA22, and miRTarBase databases (Figure 4a). Subsequently, we focused our efforts on the XIAP gene. Interestingly, XIAP was previously demonstrated to be weakly expressed in cerebral I/R, while its over-expression could protect murine neurons from cerebral I/R injury [20]. Herein, XIAP was identified as a downstream gene of miR-421 through the dual-luciferase reporter assay (*P* < 0.05, Figure 4b). Additionally, OGD/R treatment brought about marked suppression of XIAP expression in SK-N-SH cells, while the mRNA and protein levels of XIAP were augmented upon SNHG16 over-expression and reduced again upon miR-421 up-regulation (*P* < 0.05, Figure 4c). Together, these findings indicated that miR-421 inhibited the expression of XIAP.

### Silencing XIAP partly reversed the alleviating role of SNHG16 in OS injury and cell inflammation post-OGD/R

**Figure 5.**
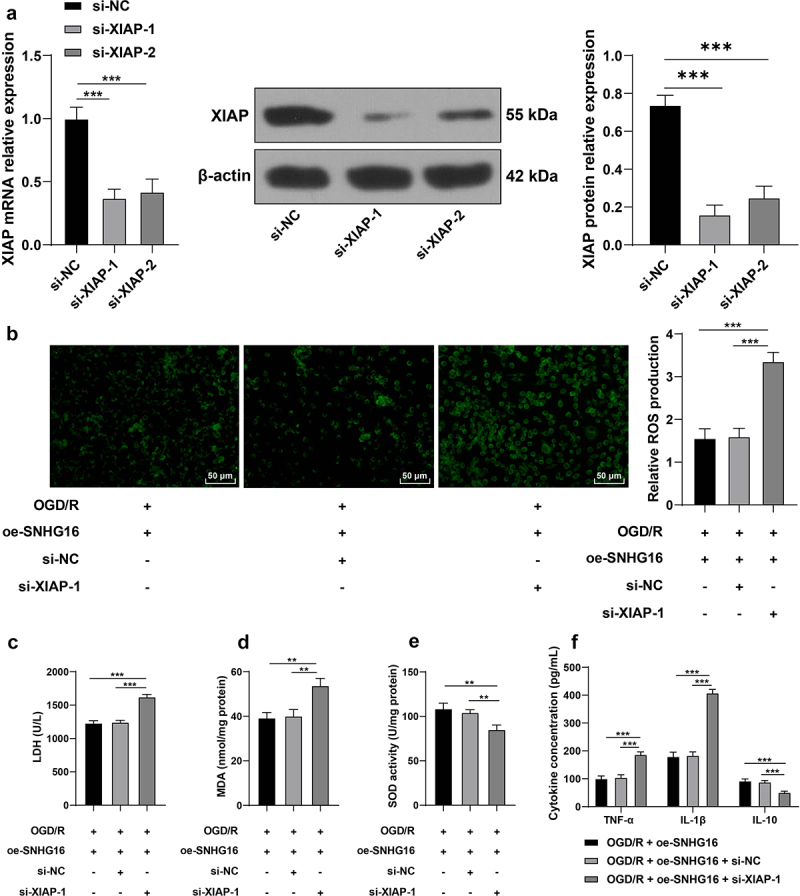
Silencing XIAP partly reversed the alleviating role of SNHG16 in OS injury and cell inflammation post-OGD/R. SK-N-SH cells were transfected with si-XIAP-1 and XIAP-2 with si-NC as the control. a: The transfection efficiency of si-XIAP-1 and XIAP-2 detected via qRT-PCR and Western blotting; si-XIAP-1 with better transfection efficacy was selected to do joint experiments with oe-SHNG15; b: ROS level measured via fluorescence labeling; c-e: LDH, MAD, and SOD activities determined via corresponding assay kits; F: TNF-α, IL-1β, and IL-10 concentrations determined via ELISA. Cell experiments were performed 3 times independently and data were exhibited as mean ± SD. Data in figures A, B, C, D, and E were analyzed using one-way ANOVA, and data in figure F were analyzed using two-way ANOVA, followed by Tukey’s multiple comparison test. * *P* < 0.05; ** *P* < 0.01; ***P < 0.001.

Lastly, in an effort to confirm the effects of XIAP on OS injury and cell inflammation post-OGD/R injury, SK-N-SH cells were transfected with si-XIAP-1 and si-XIAP-2 to down-regulate XIAP expression (*P* < 0.05, Figure 5a). Given the better transfection efficacy of si-XIAP-1, we selected si-XIAP-1 for the joint experiments with oe-SNHG16. Compared with the oe-SNHG16 group, silencing XIAP promoted ROS levels (*P* < 0.05, Figure 5b), enhanced the activity of LDH and MDA (*P* < 0.05, Figure 5c, d), weakened the SOD activity (*P* < 0.05, Figure 5e), elevated concentrations of TNF-α and IL-1β and reduced those of IL-10 (*P* < 0.05, figure 5f). Collectively, the aforementioned findings indicated that silencing XIAP partly reversed the alleviating role of SNHG16 in OS injury and cell inflammation post-OGD/R.

## Discussion

Cerebral I/R injury imposes a major clinical obstacle for physicians in the wake of ischemic stroke and is further predominantly characterized by OS injury and inflammation [36]. Interestingly, there is a plethora of evidence implicating the ceRNA network comprising lncRNA, miRNA, and mRNA, in the pathogenesis of cerebral I/R injury [37,38]. Expanding our knowledge on the same, the current study evidenced that SNHG16 competitively binds to miR-421 to promote XIAP transcription, thereby alleviating OS and inflammation post-OGD/R.

Long non-coding RNAs (lncRNAs) are well-established to play a crucial role in the adverse outcomes associated with I/R injury, such as ischemic neuronal death, inflammation, OS injury, and neurological deficits [6]. Interestingly, one such lncRNA, namely SNHG11, was previously shown to be activated by OGD/R treatment, further leading to an increased apoptosis rate of neurons [39]. In addition, prior studies have documented reduced levels of SNHG16 in neurons with I/R injury, such that SNHG16 over-expression could suppress neuronal apoptosis after cerebral I/R injury [11]. Herein, we set out with the establishment of OGD/R cell models and found that SNHG16 was down-regulated upon modeling. Additionally, OGD/R injury is known to precipitate both OS injury and inflammation, which are manifested by the over-production of ROS, LDH, MDA, TNF-α, and IL-1β and diminished release of SOD and IL-10 [40,41]. Consistently, our findings illustrated that OGD/R treatment brought about a reduction in cell proliferation, while strengthening apoptosis and inflammation and OS injury, as evidenced by increased ROS, LDH, MDA, TNF-α, and IL-1β and decreased contents of SOD and IL-10. Moreover, existing evidence suggests that SNHG16 down-regulation can facilitate hydrogen peroxide-mediated OS injury of human retinal microvascular endothelial cells *via* modulation of the miR-195/mitofusin 2 axis [42]. In a similar manner, SNHG16 possesses the ability to quench lipopolysaccharide-induced inflammation *via* sponging miR-15a/16 to promote Toll-like Receptor 4 [43]. Furthermore, prior studies have illustrated the innate potential of SNHG16 to suppress I/R injury-induced apoptosis of human brain microvascular endothelial cells [35], while also being capable of improving the survival rate of cardiomyocytes post-myocardial I/R injury [44]. In line with the aforementioned evidence, our findings indicated that over-expression of SNHG16 enhanced proliferation and inhibited apoptosis and inflammation, and OS injury caused by OGD/R treatment.

What’s more, new evidence has come to light illustrating that SNHG16 functions as a ceRNA to play roles in cerebral I/R injury by virtue of sponging its target miRNA [13]. Herein, with the help of online database prediction and nuclear/cytosol fractionation assay, we testified that SNHG16 was primarily located in the cytoplasm, underscoring that SNHG16 could indeed function as a ceRNA. Subsequently, we validated the binding relationship between miR-421 and SNHG16 *via* dual-luciferase reporter and RNA pull-down assays, such that miR-421 was suppressed by over-expression of SNHG16. In addition, the study carried out by Dong *et al*. highlighted that miR-421 could enhance neurotoxicity and neuronal death in Parkinson’s Disease [16]. More importantly, miR-421 was previously found to be aberrantly expressed in cerebral I/R injury, while also being associated with cell apoptosis and OS post-cerebral I/R injury [17]. To further explore the role of miR-421 in cerebral I/R injury, we up-regulated miR-421 in OGD/R-treated cells in the oe-SNHG16 group and uncovered that miR-421 up-regulation resulted in enhanced OS injury and aggravated inflammation, as manifested by increased ROS, LDH, MDA, TNF-α, and IL-1β concentrations and decreased SOD and IL-10 levels. Accordingly, high expressions of miR-421 were previously associated with aggravated lung inflammation in bronchopulmonary dysplasia and increased risk of inflammation in patients with metabolic syndrome [45,46]. On the other hand, another research revealed that depletion of miR-421 could alleviate hypoxia/reoxygenation-induced OS *via* up-regulation of Sirtuin-3 [47]. Moreover, miR-421 can further potentiate autophagy and apoptosis of cardiomyocytes, consequently aggravating myocardial I/R injury, which is also in accordance with our findings [48]. In lieu of these findings, it would be plausible to suggest that that miR-421 up-regulation could reverse the protecting role of SNHG16 against OS injury and inflammation in OGD/R-induced SK-N-SH cells.

Furthermore, we investigated the downstream mechanism of miR-421. Following database prediction and intersections, we focused our efforts on the expression and role of XIAP. Interestingly, XIAP is poorly-expressed in ischemic brain tissues, while also conferring a suppressive role on infarct expansion, neurological dysfunction, and neuronal apoptosis post-cerebral I/R injury [19,20,49]. The results of qRT-PCR in our study further revealed that mRNA levels of XIAP were diminished upon OGD/R treatment, elevated upon SNHG16 over-expression, and down-regulated again as a result of miR-421 up-regulation. To further evaluate the role of XIAP in cerebral I/R injury, we silenced XIAP in OGD/R-treated cells in the oe-SNHG16 group and documented that silencing XIAP countered the protective role of SNHG16 against OS injury and inflammation, as indicated by increased ROS, LDH, MDA, TNF-α, and IL-1β concentration and decreased SOD and IL-10 levels. On the contrary, over-expression of XIAP was previously correlated with neuroprotective effects, including improving neuronal angiogenesis and suppressing apoptosis, inflammation, and OS [50,51]. Consistently, there is much evidence to indicate that XIAP over-expression inhibits mitochondrial ROS production, thus protecting cells from OS injury [52]. Moreover, the alleviating role of XIAP in cerebral I/R injury is also in line with its role in intestinal and myocardial I/R injuries [53,54]. Taken in conjunction, the aforementioned findings and evidence indicate that silencing XIAP reverses the protecting role of SNHG16 against OS injury and inflammation post-OGD/R injury. To the best of our knowledge, our study is the first-of-its-kind to uncover the novel ceRNA mechanism of SNHG16 miR-421/XIAP in cerebral I/R injury.

## Conclusions

**Figure 6.**
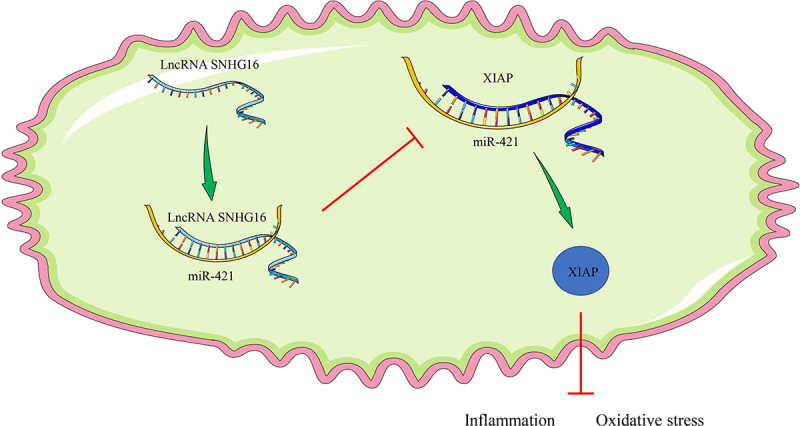
Mechanism of SNHG16 in OS injury and cell inflammation post-OGD/R. SNHG16 comparatively bound to miR-421 to inhibit the binding of miR-421 and XIAP and to further facilitate XIAP expression, thereby attenuating OS injury and cell inflammation post-OGD/R.

In summary, the current study validated the role of SNHG16 in OGD/R-treated cells *via* regulation of the miR-421/XIAP axis (Figure 6). However, we solely explored the downstream mechanism of SNHG16 with the focuses on miR-421 and XIAP, while failing to discuss other downstream targets of SNHG16. Additionally, our study failed to investigate the upstream mechanism of SNHG16 and its effects in animal and clinical models. To augment the transition of this theory into clinical application, it would be prudent to explore more downstream targets and carry out animal experiments in combination with clinical analyses in future studies.

## Data Availability

The data and materials that support this study are available from the corresponding author upon reasonable request.

## Research highlights

- SNHG16 is poorly expressed in OGD/R-treated cells.
- SNHG16 over-expression suppresses OS and cell inflammation in OGD/R-treated cells.
- SNHG16 competitively binds to miR-421 to promote XIAP expression.
- miR-421 up-regulation reverses the protecting role of SNHG1 in OGD/R-treated cells.
- SNHG16 alleviates OS and cell inflammation *via* the miR-421/XIAP axis.
